# A Novel Loss-of-Function Mutation in the NPRL3 Gene Identified in Chinese Familial Focal Epilepsy with Variable Foci

**DOI:** 10.3389/fgene.2021.766354

**Published:** 2021-11-12

**Authors:** Youzhi Li, Xu Zhao, Shanshan Wang, Ke Xu, Xin Zhao, Shanshan Huang, Suiqiang Zhu

**Affiliations:** ^1^ Department of Neurology, Tongji Hospital of Tongji Medical College, Huazhong University of Science and Technology, Wuhan, China; ^2^ Department of Radiology, Tongji Hospital of Tongji Medical College, Huazhong University of Science and Technology, Wuhan, China

**Keywords:** GATOR1, Nprl3, mTOR signaling pathway, whole-exome sequencing (WES), familial focal epilepsy with variable foci

## Abstract

Familial focal epilepsy with variable foci is an autosomal dominant disorder characterized by partial epilepsy with variable foci. In this study, we report a six-generation with segregation of the mutation present in four generations Chinese family presenting with focal epilepsy with variable foci. Whole exome sequencing confirms a novel pathogenic mutation in the NPRL3 gene (c316C>T; *p*. Q106*). PCR, Western blotting, and immunohistochemistry were conducted to analyze the gene transcription, protein expression, and subcellular localization of NPRL3 and related signaling molecules in peripheral blood cells from family members. As compared with healthy family members, both mRNA level and protein expression of NPRL3 are decreased in peripheral blood cells of the mutation carrier. In addition, the expression of downstream molecular Phospho-p70 S6 kinase (P-s6k) are increased consequently. Our findings expand the genotypic and phenotypic spectrum of the NPRL3-associated epilepsy and reveal the mechanisms of mTOR pathway signaling and GATOR1 pathogenesis in focal epilepsies, providing exciting potential for future diagnostic and therapeutic interventions. However, further *in vitro* and animal experiments are still needed to evaluate the role of NPRL3 loss-of-function mutation in epileptogensis.

## Introduction

Familial focal epilepsy with variable foci (FFEVF, OMIM 604364), also named familial partial epilepsy with variable foci (FPEVF), is an autosomal dominant disorder characterized by partial epilepsy with variable foci. It was initially described in an Australian family with focal epilepsies arising from different lobes, including the frontal, temporal, frontotemporal, parietal, and occipital cortical regions, among the affected family members (Scheffer et al., 1998) though most of the patients have their epileptic focus in the frontal or temporal lobe (Lu et al., 2008). Focal to bilateral tonic-clonic seizures (FBTCS) may occur with the spreading discharge. Sex ratio (male/female) of FPEVF is 0.8–1.5. The onset of seizure ranges from infants (1-month-old) to adults (43 years old), and the median age is 13 years with the peak at 5 and 25. Structural magnetic resonance imaging (MRI) and computed tomography (CT) usually do not reveal unremarkable changes in the brains of FFEVF, but single photon emission computed tomography (SPECT) could show hypertransfusion of the associated seizure regions (Picard et al., 2000). Families with FFEVF show an autosomal dominant inheritance pattern with incomplete penetrance. To date, mutations in the DEP domain (Dishevelled, Egl-10, and Pleckstrin domain)-containing protein 5 (*DEPDC5*, NM_001242896.1) genes were identified as the most common cause for FFEVF (Ishida et al., 2013). However, because FFEVF presents a wide spectrum of clinical manifestations, more investigation needs to identify additional genetic causes of this disease.

Recently, an increasing number of presumably pathogenic mutations in GATOR1 complex genes were reported in cases of focal epilepsy (Iffland et al., 2019). The GATOR1 complex consists of the nitrogen osmosis regulatory factors-3 (*NPRL3*, NM_001077350.2), nitrogen osmosis regulatory factors -2 (*NPRL2* ,NM_006,545.4), and *DEPDC5*, which are involved in the mTOR cascade reactions (Bar-Peled et al., 2012). To date, around 80 different *NPRL3* mutations were found to associate with sporadic and familial general epilepsy as well (Carvill et al., 2015; Baldassari et al., 2019). In addition, several epileptic syndromes, such as nocturnal frontal lobe epilepsy (NFLE) and benign epilepsy in children with centro-temporal spikes, were also associated with *NPRL3* (Carvill et al., 2015; Baldassari et al., 2016; Korenke et al., 2016; Baldassari et al., 2019). Notably, numerous mutations in the coding region of the *NPRL3* gene have so far been reported in FFEVF families. Exome sequencing analysis identified three *NPRL3* mutations (c.835_836insT, *p*. Ser279Phe fs*52 c.314T>C, *p*. Leu105Pro) in multiplex families with focal epilepsy originated in both temporal and frontal lobes, respectively (Ricos et al., 2016). Two *NPRL3* (c.1270C>T, *p*. Arg424*, c.1070delC, *p*. Pro357Hisfs*56) mutations were also identified in FFEVF families (Weckhuysen et al., 2016). Christina Canavati and colleagues detected an *NPRL3* mutation (c.1063C>T, *p*. Gln355*; ∼ 38-kb deletion encompassing eight exons 8–15) in Palestinian FFEVF families (Canavati et al., 2019).

In this study, we examined a six-generation family with segregation of the mutation present in four generations with FFEVF and identified a new genetic mutation, c316C>T/*p*. Q106*, in the *NPRL3* gene, expanding the phenotypic spectrum of *NPRL3*-associated epilepsy. Furthermore, the functional study revealed that the new identified *NPRL3* mutations significantly affected the transcript expression of NPRL3, resulting in hyperactivation of the mTOR signaling pathway.

## Materials and Methods

### Patients

**FIGURE 1:**
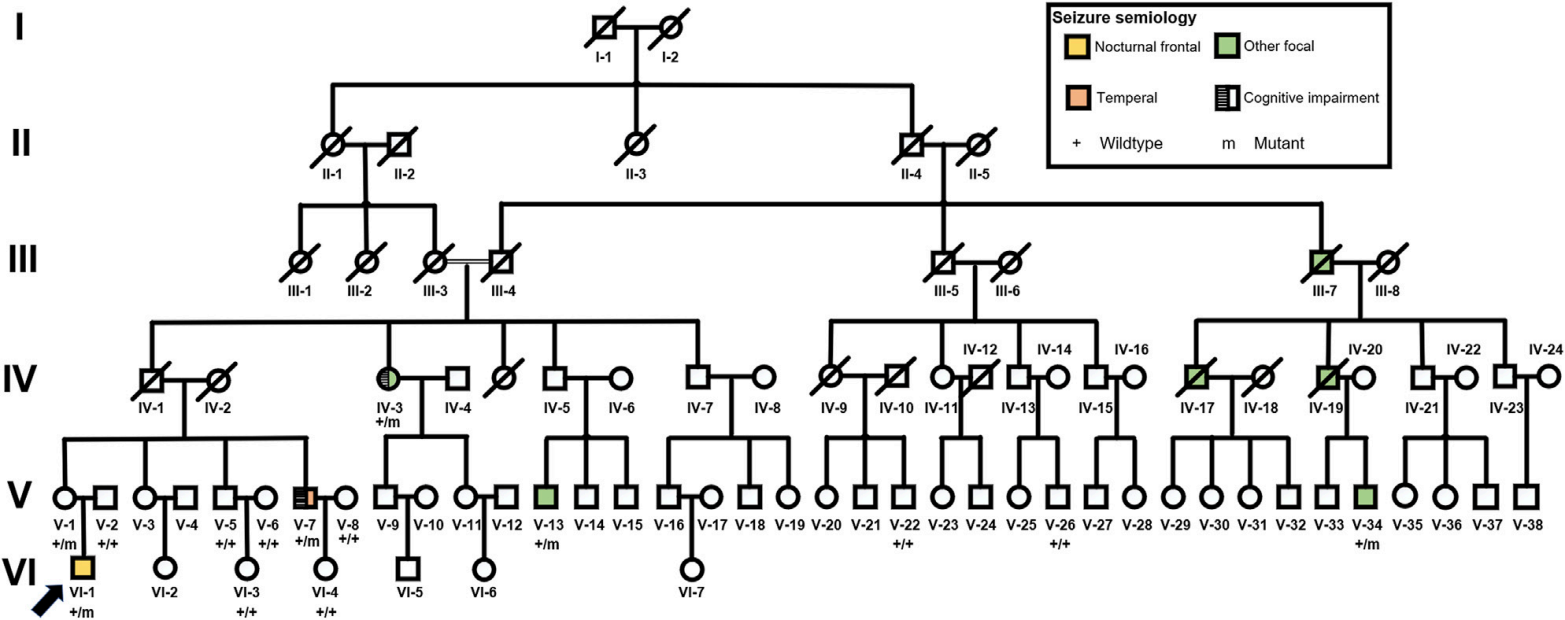
Pedigree of the FFEVF with the NPRL3 *p*. Gln106* mutation.

The autosomal dominant (AD) FFEVF family with seven affected individuals (Figure 1) was recruited in the department of neurology (Tongji hospital, WuHan). After obtaining permission to have access to the affected members’ medical records, clinical manifestation, including epilepsy symptomology, electroencephalogram (EEG), MRI, and any other neurological disorders related to epilepsy diagnosis, were collected for analysis. Additional EEGs were performed in individuals V-7, IV-3, V-13, and V-34 and MRI in IV-3, V-13, and V-34. Family members underwent phenotyping by filling in a seizure questionnaire, review of clinical investigation, and accessory examinations. Epilepsy was classified according to current recommendations of the International League against epilepsy revised organization. Peripheral blood cells were collected from individuals IV-3, V-1, V-2, V-7, V-13, VI-1, V-5, V-6, V-7, V-8, V-22, V-26, VI-3, and VI-4 using standard procedures.

### Genotyping

DNA samples were prepared from peripheral blood cells of family members using a Blood DNA Extraction Kit (Vazyme Biotech Co., Nanjing, China). Whole exome sequencing was applied in five affected individuals IV-3, V-1, V-2, V-7, and V-13 and three unaffected individuals VI-1, V-22, and V-26. Exomes were captured using a VAHTS^®^ mRNA-seq v3 Library Prep Kit (Vazyme Biotech Co., Nanjing, China). Sequencing was performed on an Illumina HiSeq X Ten instrument (Illumina Inc., San Diego, CA). Picards was used to remove redundant PCR amplification products. A Genome Analysis Toolkit was used to detect and filter InDel and SNPs. The Annovar linked gene database includes 1000 genomes, the Exome Aggregation Consortium (EXAC). After the relevant mutation data were obtained, the genomic reference sequence of the relevant mutation was obtained from NCBI Gene, followed by Sanger sequencing to verify the result. Additionally, genotyping was applied by Sanger sequencing in healthy family members V-5, V-6, V-8, VI-3, and VI-4 and co-segregation analysis. The forward primer for NPRL3 was GTG​CAG​TTT​ACT​GAG​ACC​CTT​CCG; the reverse primer was GCC​CAG​GGG​AAG​CCA​AAT​CTA​C.

### *In Silico* Analysis

The pathogenicity of gene mutation was predicted by mutation taster, SIFT (http://sift.jcvi.org/), CADD, and PolyPhen-2 (http://genetics.bwh.harvard.edu/pph2/). All the nonsynonymous, nonsense, frameshift, and splice-site variants with a minor allele frequency <1% in ExAC (http://exac.broadinstitute.org) and gnomAD (https://gnomad.broadinstitute. org) databases were included. The clinical interpretation of the genetic mutation was based on The American College of Medical Genetics and Genomics (ACMG)and the Association for Molecular Pathology (AMP) 2015 guideline.

### *In Vitro* Experiments

Using percoll density gradients for leukocyte isolation (Ulmer et al., 1984), peripheral blood cells (PBCs) of individuals IV-3, V-13, and V-1 carrying the *NPRL3* gene mutation (c.316C>T, *p*. Gln106*) and three age-matched healthy people and healthy family members V-2, V-22, and V-26 were collected. Total RNA from PBCs was extracted by the TRIzol method (Sigma-Aldrich, Darmstadt, Germany) and reverse transcribed with HiScript III qRT SuperMix (Vazyme Biotech Co., Nanjing, China), and the qPCR System was applied with ChamQ SYBR qPCR Master Mix (Vazyme Biotech Co., Nanjing, China) on a CFX96 Touch Real-Time PCR Detection System (bio-rad, California, USA). NPRL3 cDNA was amplified using specific primers located inside the deleted region. The forward primer was CAG​CTC​ATC​TGG​TGT​ACT​GGG; the reverse primer was GGG​GCC​AGG​GGA​TTC​CTA​AA. Histochemistry and Western blot was performed to assess the NPRL3 protein expression level (Anti-Mare antibody, Abcam #ab121346; 1:100 dilution) and mTORC1 activity (S6k1 Rabbit mAb, Cell Signaling #4856; 1:200 dilution; S6k1 Rabbit mAb, Abcam #ab32359; 1:200 dilution), and slides were observed with an OLYMPUS BX51 fluorescence microscope (OLYMPUS, Tokyo, Japan).

## Results

### Clinical Description of FFEVF Family

There were eight patients with seizure out of 84 family members in this six-generation Chinese family with segregation of the mutation present in four generations with FFEVF (Figure 1). The NPRL3 mutations had a calculated penetrance of 50% or less in our family. All the patients in the family had no history of febrile seizure and specific brain abnormality although two had mild cognitive impairment. Age at onset of seizures ranged from 8 to 20 years (median 9 years). Two patients have nocturnal seizures, and one had diurnal seizures only, and in the other two, sleep-related seizures predominated. Epilepsy types range from frontal to temporal lobe epilepsy, and all patients manifested FBTCS type. Seizures were preceded by emotional aura and sensory auras, including fear and headache. Duration of seizures ranged from 1 to 5 min. The frequency of attacks ranged from weekly to monthly, and the frequency and severity of attacks decreased with age even when the patient was refractory to ASMs. Most of the patients had a good response to phenobarbital and carbamazepine; however, three have ongoing seizures. The clinical features of the five patients available for clinical evaluation are included in Table 1.

**TABLE 1:** Clinical features of the patients in the FFEVF family.

Subject,sex	Age at onset (years)	Age studied (years)	Time of seizure	Phenotype	Seizure type	ASM use	EEG	MRI	Seizure outcome
IV-3, F	8	74	Diurnal	Unclassified	FBTCS	PB	Normal	Brain atrophy	Seizure-free
V-7, M	16	42	Nocturnal, diurnal	TLE	FBTCS	Clonazepam, PTH, VPA	Sharp and slow wave complex predominantly in right temporal lobe	Normal	10 seizures per year
V-13, M	9	41	Nocturnal	Unclassified	FBTCS	PB	Normal	Normal	10 epileptic auras per year
V-34, M	20	37	Nocturnal, diurnal	Unclassified	FBTCS	PB, CBZ	Normal	Normal	1–2 seizures per year
VI-1, M	19	28	Nocturnal	FLE	FBTCS	TGM	Sharp waves in the right frontal lobe; normal later	Normal	Seizure-free

The proband (VI-1, 28 years) started experiencing (FBTCS) at the age of 19 during sleep with no diurnal seizure (Figure 1; Table 1). Seizure attacks initiated with head version and tonic–clonic movements of the left arm and then progressed to bilateral tonic–clonic movements. Interictal EEG showed bilateral 5- to 7-Hz *θ* waves and sporadic sharp waves primarily in the right frontal lobe (Supplementary Figure S1A). The 1.5-tesla (1.5T) MRI did not reveal brain abnormalities. He had one to two seizures per month and was seizure free at the age of 21 on lamotrigine. He is developing normally.

The proband’s uncle (V-7, 42 years) experienced his first seizure at the age of 16 with predominant nocturnal seizures. The FBTCS started with motor automatisms presented as shouting and tonic movements of the lower limbs and then progressed to bilateral tonic–clonic movements lasting 2–3 min. Long-term electroencephalographic monitoring (LTM) showed 1.5- to 2-Hz sharp and slow wave complex predominantly in the right temporal lobe (Supplementary Figure S1B). The 3T MRI did not reveal brain abnormalities. Seizure frequency was two to three times weekly and decreased to 10 times per year on valproate and phenytoin. He was diagnosed with mild cognitive impairment as the score of Mini-Mental State Examination (MMSE) was 23 at the age of 41.

The proband’s grandaunt (IV-3, 74 years) developed FBTCS at 8 years old. She presented with a similar seizure to patient V-7 except for an inexplicable fear aura. LTM showed a normal record, and 3 T MRI showed age-related brain atrophy. Seizure frequency was three to four times per year. She responded well to phenobarbital and was seizure free at the age of 72. She showed mild cognitive impairment with a MMSE score of 21 at the age of 74.

The uncle of the proband (V-13, 41 years) had a similar seizure to the proband at the age of 9. He occasionally experienced atonic movements, which progressed to bilateral tonic–clonic movements. Seizures were preceded by a sensory aura, which presented as headache. EEG and MRI were normal. Seizure frequency was seven to eight times monthly at the age of 9 and decreased to 10 times per year after phenobarbital treatment. He is developmentally normal.

The proband’s uncle (V-34, 37 years) presented with FBTCS at age 20 years. The patient experienced bilateral tonic–clonic movements occasionally preceded by shouting. He had unbearable post-ictal headache with a pain score of seven points. EEG and MRI were normal.Seizure frequency was one to two times monthly at age 20 and decreased to one to two times per year at the age of 37 on phenobarbital and carbamazepine. He is developmentally normal.

#### *NPRL3* mutations

**FIGURE 2:**
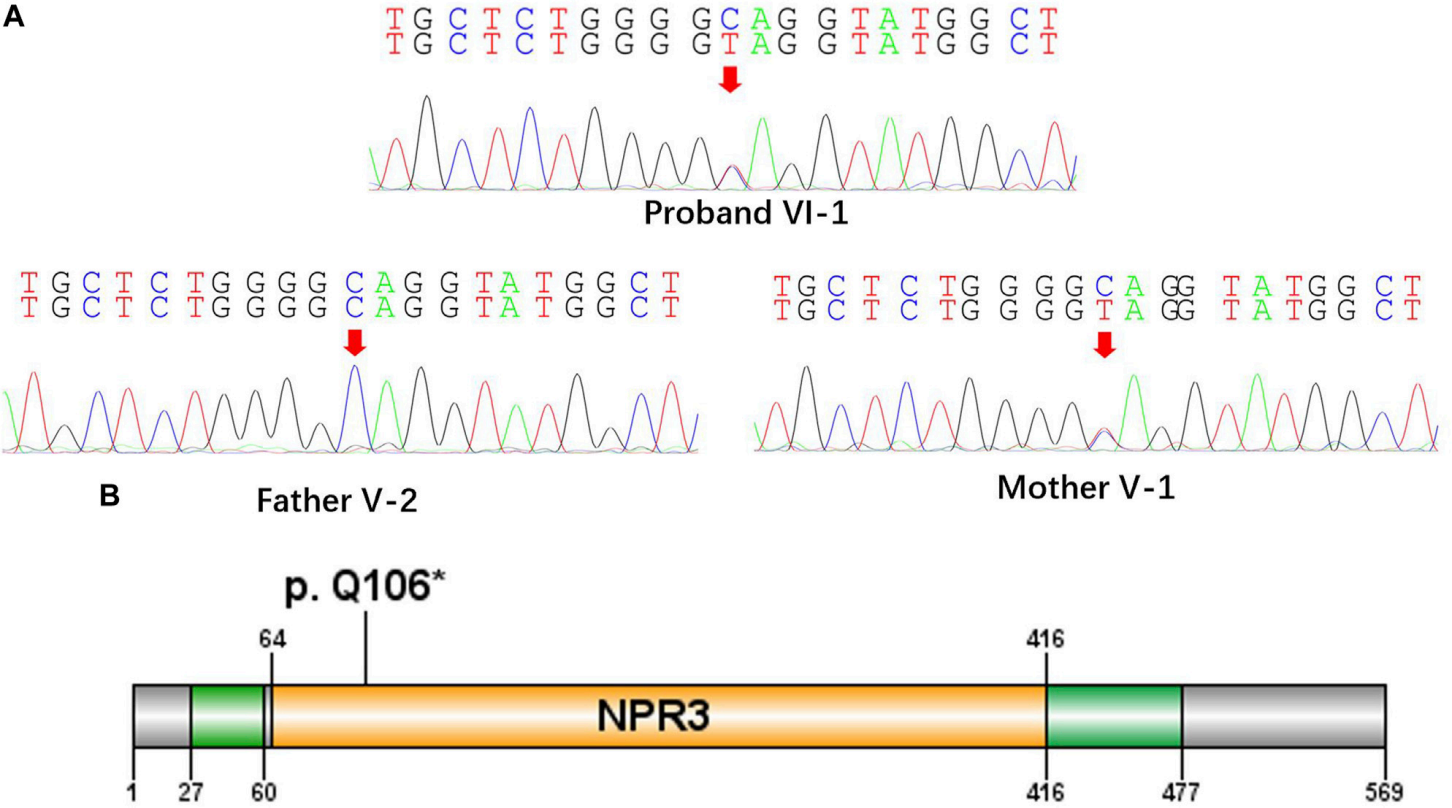
A nonsense mutation c.316C>T, *p*. Gln106* in the NPRL3 gene. **(A)** Sanger sequencing of the parent-proband trio (patients VI-1, V-1, and V-2). The filled symbol indicates the affected individual. **(B)** Protein schematic of NPRL3.

All the DNA samples passed the quality control measures. For WES, around 9–11 GB of clean reads were obtained from each sample, and more than 99% of the measured bases were aligned to the genome reference consortium (hg19). The average sequencing depth is 146.86-fold, and a 99% average read depth of the target regions were covered at 10x, and 98% at 20x. Following bioinformatics analysis of sequencing data, we identified a novel *NPRL3* gene mutation (c.316C>T, *p*.Gln106*) in an autosomal dominant (AD) six-generation family with focal epilepsy by WES. All of the identified mutations were confirmed by Sanger sequencing (Figure 2). The *NPRL3* gene mutation (c.316C>T) was co-segregated with the disease, and all the affected members among IV-3, V-1, V-2, V-7, V-13, VI-1, V-5, V-6, V-7, V-8, V-22, V-26, VI-3, and VI-4 had the mutation, and all the healthy members did not carry the mutation except for V-1. *In silico* analysis showed the *NPRL3* gene (c.316C>T) mutation was disease causing through the proposed mechanism of nonsense mutation-mediated mRNA decay. The ACMG 2015 guideline classified the *NPRL3* gene (c.316C>T) mutation into likely pathogenic (PVS1, PM2, PP1, PP4).

### Molecular Studies

**FIGURE 3:**
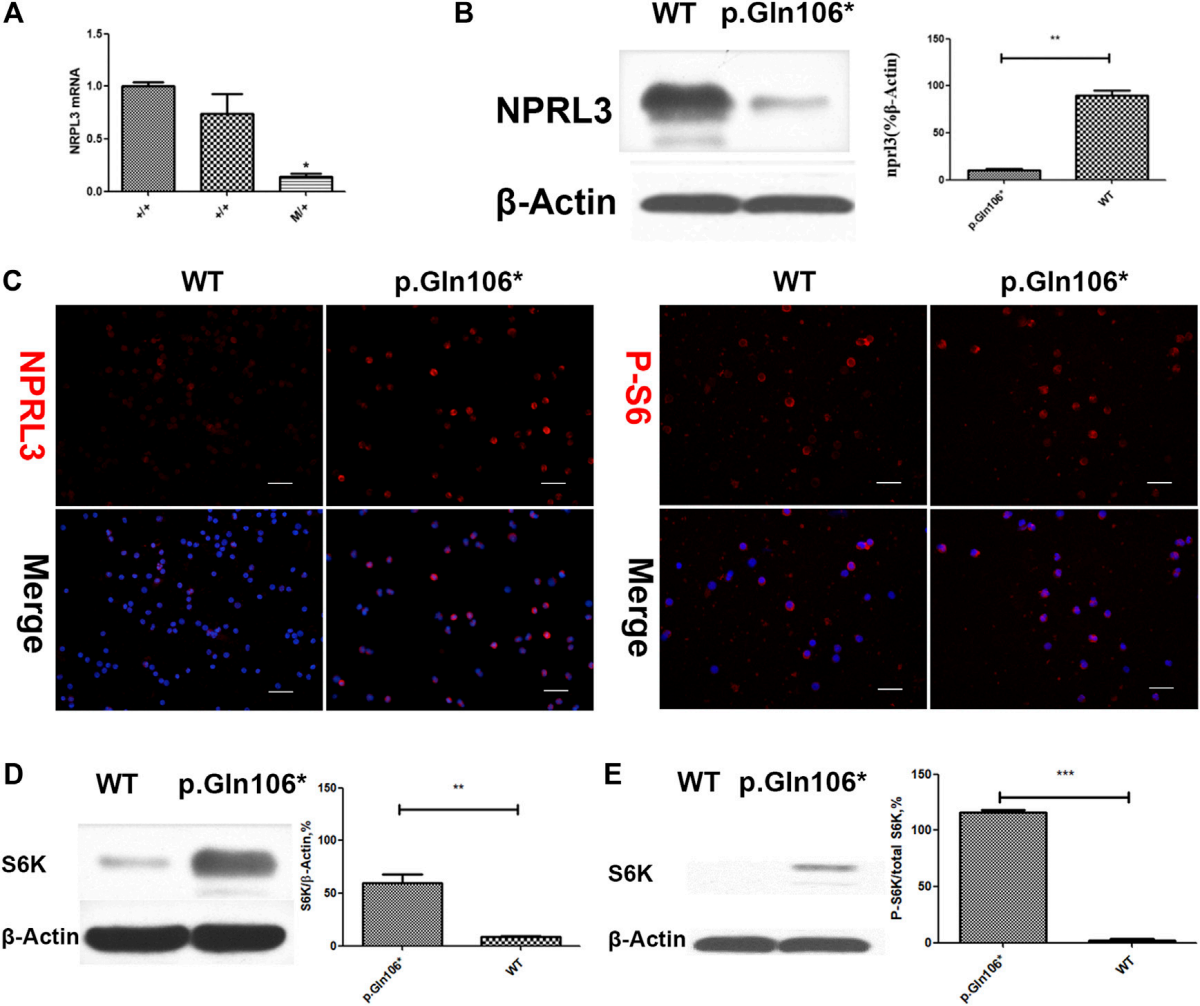
Analysis of steady-state mRNA levels in peripheral blood cells from individuals with mutations in NPRL3 predicted to result in haploinsufficiency. Group p. Gln106* included IV-3, V-13, and V-1 carrying *NPRL3* gene mutation (c.316C>T, *p*. Gln106*) and group WT included three healthy family members V-2, V-22, and V-26. **(A)** Real-time (RT) quantitative polymerase chain reaction (PCR) analysis of wild-type (+/+) and mutation carrier (m/+) RNA was performed, and the transcription of NPRL3 was significantly reduced in the group of mutation carriers (*p < 0.05). The experiment was repeated at least three times and produced consistent results. **(B–E)** Western blot and immunobiology showed the expression of NPRL3 was decreased and the expression of PS6 was increased in peripheral blood lymphocytes of patients with P.Gln106 * mutation。.

Analysis of RNA and protein from the peripheral blood cells demonstrated decreased *NPRL3* transcription and expression in the mutation carriers (Figure 3A,B). Phospho-S6 ribosomal immunostaining was positive in peripheral blood cells of the *NPRL3* mutation carrier (Figure 3C–E), consistent with mTOR pathway activation.

## Disscussion

We report a six-generation family with segregation of the mutation present in four generations with FFEVF, and genetic investigation revealed a nonsense mutation c.316C>T, *p*. Gln106* in the *NPRL3* gene segregating with FFEVF in an autosomal dominant pattern, which is a novel mutation absent in ExAC and gnomAD databases. mTORopathies are neurodevelopmental disorders that are caused by loss-of-function (LOF) mutations in negative regulators of mTORC1 (Liu and Sabatini, 2020). A unique subset of mTORopathies caused by GATOR1 complex mutations was subclassified as “GATORopathies.” Almost two thirds of the GATORopathies are composed of sleep-related hypermotor seizures and focal epilepsies, and FFEVF patients are only reported in less the 10% of cases (Baldassari et al., 2019). The report of a novel familial mutation in NPRL3 presenting with FFEVF is of certain significance in understanding the disease.

As a familial focal epilepsy with an autosomal dominant pattern, the possible diagnoses were sleep-related hypermotor epilepsy (SHE), autosomal dominant epilepsy with auditory features (ADEAF), and familial focal epilepsy with variable foci (FFEVF) (Kaur, 2013). SHE, formerly known as nocturnal frontal lobe epilepsy (NFLE), is characterized by clusters of nocturnal motor seizures and is associated with mutations in ligand-gated neuronal nicotinic acetylcholine receptor (nAChR) (Scheffer, 2000). Remarkably, DEPDC5 LOF mutations were also found in 13% of the families with a presentation of autosomal dominant nocturnal frontal lobe epilepsy (ADNFLE) (Picard et al., 2014). ADNFLE and FFEVF have genotypical and phenotypical overlap, so it is important to exclude the ADNFLE diagnosis in families present with nocturnal seizures. For SHE, all affected individuals in the family must have seizures compatible with sleep-related hypermotor movements. A predominance of awake seizures is also reported to be a useful distinction between FFEVF and SHE (Tinuper et al., 2016). Autosomal dominant epilepsy with auditory features (ADEAF) is a focal epilepsy syndrome with familial lateral temporal lobe seizure in affected family members, and is characterized with auditory auras and aphasic seizures (Klein et al., 2016). In our series, VI-1 had nocturnal frontal lobe seizures, IV-3 had only awake seizures, and V-7 had temporal lobe seizures, favoring a diagnosis of FFEVF. There are two of five patients with mild cognition impairment in the FFEVF family. Cognition decline is the most common comorbidity of epilepsy, which is prevalent in mTORopathy patients (Nickels et al., 2016). A cohort including 73 probands showed that 44% of the probands had neurocognitive deficits (Baldassari et al., 2019). In addition, GATOR1-related genes play vital roles in brain development (Bockaert & Marin, 2015). Therefore, repeated seizures and genetically driven pathological changes might all contribute to the cognitive decline for our patients.

Studies of other mTORopathies suggest that GATOR1 and mTORC1 signaling are likely to be involved in embryonic neurogenesis as well as signaling in the adult brain (Baulac, 2016). NPRL3 can affect cell structure and signal transduction through the mTORC1 pathway and also affects cell polarity and the growth process during development (Iffland et al., 2018). Increased cell volume and PS6 activity were found in brain samples of FCD type Ia patients with *NPRL3* LOF mutations *NPRL3* (Sim et al., 2016). The same change was observed in the *NPRL3* shRNA silenced Neuro2A cells. Besides this, the *NPRL3* knockout permeates the co-localization of mTOR and the lysosomal membrane under the condition of amino acid starvation, and all of these changes could be reversed after the application of rapamycin (Iffland et al., 2018). The functional loss of the *NPRL3* gene may cause the abnormal development of filamentous pseudo and dendrites, leading to the abnormal localization of neurons, and finally causing the structural abnormality of the cerebral cortex in FCD (Baldassari et al., 2016).

The proposed pathogenic mechanism of the NPRL3 gene mutation is that LOF causes an haploinsufficiency induced by nonsense-mediated decay of mRNA as previously suggested that truncating mutations in *NPRL3* reduce transcript levels significantly (Canavati et al., 2019). In this study, we found a nonsense mutation c.316C>T, *p*. Gln106* in the *NPRL3* gene, which is located in exon4 (Figure 2). By analyzing the NPRL3-mTOR signaling pathway in family members *in vivo*, we confirmed the epilepsy was associated with decreased NPRL3 protein expression levels and increased mTOR pathway activity. Moreover, mTOR activity was increased in PBCS of the FFEVF patients, indicating a ubiquitous hyperactivation of the mTOR signaling pathway in *NPRL3* mutation carriers. Our results further emphasize the role of aberrant regulation of mTOR signaling in *NPRL3* mutation–related seizures and *NPRL3* LOF in the genesis of epilepsy.

It is interesting to note that the *NPRL3* mutation in this family is not of full penetrance. The possible explanation for the marked phenotypic variability is that the epigenetic regulation and the second hit during brain development may greatly influence the severity of the phenotypes. *NPRL3* In the previous studies, *NPRL3* gene mutations are reported to be the likely cause for malformation of cortical development (MCDs) and MCD-related epilepsy (Iffland et al., 2019). However, our results suggest that the *NPRL3* gene mutations cannot be excluded in MRI-normal epilepsy patients.

Existing mTORC1 inhibitors, such as rapamycin, show promise in the treatment of focal epilepsy associated with tuberous sclerosis and might also be effective for other focal epilepsy syndromes (Myers and Scheffer, 2017). The presumable mechanism of treatment is thought to be the inhibition of formation and growth of cortical tubers that are presumed to be epileptogenic. Another hypothesis is that rapamycin and analogs can suppress the inflammation reaction by increasing IL-1β and other inflammatory cytokines and chemokines in the neocortex and hippocampus (Zhang et al., 2015) mTORC1 antagonists in the treatment of focal epilepsy are promising, especially for individuals carrying mutations of GATOR1 genes, including DEPDC5, NPRL2, and NPRL3. Our study has several limitations. The inability to obtain brain tissue samples from the family members prevented us from obtaining more evidence for altered NPRL3 expression in the responsible brain region, and a PET scan is needed to reveal the subtle metabolic or biochemical function changes in the brain of the FFEVF family patients.

## Conclusion

To our knowledge, this is a novel nonsense mutation of NPRL3 found in an FFEVF family. Our results reveal the mechanisms of mTOR pathway signaling and GATOR1 pathogenesis in focal epilepsies. The detection of an increasing number of patients with GATOR1 complex mutations and further investigations into the mechanisms of mTOR pathway signaling and GATOR1 pathogenesis in focal epilepsies present exciting potentials for future therapeutic interventions.

## Data Availability

The datasets presented in this study can be found in online repositories. The names of the repository/repositories and accession number(s) can be found below:ClinVar, SCV001934196.

## Abbreviations

ASM, antiseizure medication; CBZ, carbamazepine; EEG, electroencephalogram; F, female; FLE, frontal lobe epilepsy; FBTCS, focal to bilateral tonic–clonic seizure; LTG, lamotrigine; M, male; MRI, magnetic resonance imaging; PB, phenobarbital; PHT, phenytoin; TLE, temporal lobe epilepsy; VPA, valproic acid.

## References

[B1] Baldassari S. Licchetta L. Tinuper P. Bisulli F. Pippucci T. (2016). GATOR1 Complex: the Common Genetic Actor in Focal Epilepsies. J. Med. Genet. 53 (8), 503–510. 10.1136/jmedgenet-2016-103883 27208208

[B2] Baldassari S. Picard F. Verbeek N. E. van Kempen M. Brilstra E. H. Lesca G. (2019). The Landscape of Epilepsy-Related GATOR1 Variants. Genet. Med. 21 (2), 398–408. 10.1038/s41436-018-0060-2 30093711 PMC6292495

[B3] Bar-Peled L. Schweitzer L. D. Zoncu R. Sabatini D. M. (2012). Ragulator Is a GEF for the Rag GTPases that Signal Amino Acid Levels to mTORC1. Cell 150 (6), 1196–1208. 10.1016/j.cell.2012.07.032 22980980 PMC3517996

[B4] Baulac S. (2016). mTOR Signaling Pathway Genes in Focal Epilepsies. Prog. Brain Res. 226, 61–79. 10.1016/bs.pbr.2016.04.013 27323939

[B5] Bockaert J. Marin P. (2015). mTOR in Brain Physiology and Pathologies. Physiol. Rev. 95 (4), 1157–1187. 10.1152/physrev.00038.2014 26269525

[B6] Canavati C. Klein K. M. Afawi Z. Pendziwiat M. Abu Rayyan A. Kamal L. (2019). Inclusion of Hemimegalencephaly into the Phenotypic Spectrum of NPRL 3 Pathogenic Variants in Familial Focal Epilepsy with Variable Foci. Epilepsia 60 (6), e67–e73. 10.1111/epi.15665 31111464

[B7] Carvill G. L. Crompton D. E. Regan B. M. McMahon J. M. Saykally J. Zemel M. (2015). Epileptic Spasms Are a Feature ofDEPDC5mTORopathy. Neurol. Genet. 1 (2), e17. 10.1212/NXG.0000000000000016 27066554 PMC4807908

[B8] Iffland P. H. 2nd Baybis M. Barnes A. E. Leventer R. J. Lockhart P. J. Crino P. B. (2018). DEPDC5 and NPRL3 Modulate Cell Size, Filopodial Outgrowth, and Localization of mTOR in Neural Progenitor Cells and Neurons. Neurobiol. Dis. 114, 184–193. 10.1016/j.nbd.2018.02.013 29481864 PMC6413529

[B9] Iffland P. H. 2nd Carson V. Bordey A. Crino P. B. (2019). GATOR Opathies: The Role of Amino Acid Regulatory Gene Mutations in Epilepsy and Cortical Malformations. Epilepsia 60 (11), 2163–2173. 10.1111/epi.16370 31625153 PMC7155771

[B10] Ishida S. Picard F. Rudolf G. Noé E. Achaz G. Thomas P. (2013). Mutations of DEPDC5 Cause Autosomal Dominant Focal Epilepsies. Nat. Genet. 45 (5), 552–555. 10.1038/ng.2601 23542701 PMC5010101

[B11] Kaur A. (2013). NovelDEPDC5mutations Causing Familial Focal Epilepsy with Variable Foci Identified. Clin. Genet. 84 (4), 341–342. 10.1111/cge.12239 23869883

[B12] Klein K. M. Pendziwiat M. Cohen R. Appenzeller S. de Kovel C. G. F. Rosenow F. (2016). Autosomal Dominant Epilepsy with Auditory Features: a New LGI1 Family Including a Phenocopy with Cortical Dysplasia. J. Neurol. 263 (1), 11–16. 10.1007/s00415-015-7921-2 26459092

[B13] Korenke G.-C. Eggert M. Thiele H. Nürnberg P. Sander T. Steinlein O. K. (2016). Nocturnal Frontal Lobe Epilepsy Caused by a Mutation in the GATOR1 Complex geneNPRL3. Epilepsia 57 (3), e60–e63. 10.1111/epi.13307 26786403

[B14] Liu G. Y. Sabatini D. M. (2020). mTOR at the Nexus of Nutrition, Growth, Ageing and Disease. Nat. Rev. Mol. Cel Biol 21 (4), 183–203. 10.1038/s41580-019-0199-y PMC7102936 31937935

[B15] Lu Y. Yu W. Shen D. Wang X. (2008). Three New Forms of Familial Epilepsy Syndromes in the Proposed Diagnostic Scheme of the ILAE (2001): a Clinical Experience in Southwest China. Epilepsia 49 (6), 1103. 10.1111/j.1528-1167.2008.01549_1.x 18554358

[B16] McDaniel S. S. Rensing N. R. Thio L. L. Yamada K. A. Wong M. (2011). The Ketogenic Diet Inhibits the Mammalian Target of Rapamycin (mTOR) Pathway. Epilepsia 52 (3), e7–e11. 10.1111/j.1528-1167.2011.02981.x 21371020 PMC3076631

[B17] Myers K. A. Scheffer I. E. (2017). DEPDC5 as a Potential Therapeutic Target for Epilepsy. Expert Opin. Ther. Targets 21 (6), 591–600. 10.1080/14728222.2017.1316715 28406046

[B18] Nickels K. C. Zaccariello M. J. Hamiwka L. D. Wirrell E. C. (2016). Cognitive and Neurodevelopmental Comorbidities in Paediatric Epilepsy. Nat. Rev. Neurol. 12 (8), 465–476. 10.1038/nrneurol.2016.98 27448186

[B19] Picard F. Baulac S. Kahane P. Hirsch E. Sebastianelli R. Thomas P. (2000). Dominant Partial Epilepsies: A Clinical, Electrophysiological and Genetic Study of 19 European Families. Brain 123 (Pt 6), 1247–1262. 10.1093/brain/123.6.1247 10825362

[B20] Picard F. Makrythanasis P. Navarro V. Ishida S. de Bellescize J. Ville D. (2014). DEPDC5 Mutations in Families Presenting as Autosomal Dominant Nocturnal Frontal Lobe Epilepsy. Neurology 82 (23), 2101–2106. 10.1212/WNL.0000000000000488 24814846

[B21] Ricos M. G. Hodgson B. L. Pippucci T. Saidin A. Ong Y. S. Heron S. E. (2016). Mutations in the Mammalian Target of Rapamycin Pathway regulatorsNPRL2andNPRL3cause Focal Epilepsy. Ann. Neurol. 79 (1), 120–131. 10.1002/ana.24547 26505888

[B22] Scheffer I. E. (2000). Autosomal Dominant Nocturnal Frontal Lobe Epilepsy. Epilepsia 41 (8), 1059–1060. 10.1111/j.1528-1157.2000.tb00298.x 10961640

[B23] Scheffer I. E. Phillips H. A. O'Brien C. E. Saling M. M. Wrennall J. A. Wallace R. H. (1998). Familial Partial Epilepsy with Variable Foci: a New Partial Epilepsy Syndrome with Suggestion of Linkage to Chromosome 2. Ann. Neurol. 44 (6), 890–899. 10.1002/ana.410440607 9851433

[B24] Sim J. C. Scerri T. Fanjul-Fernández M. Riseley J. R. Gillies G. Pope K. (2016). Familial Cortical Dysplasia Caused by Mutation in the Mammalian Target of Rapamycin regulatorNPRL3. Ann. Neurol. 79 (1), 132–137. 10.1002/ana.24502 26285051

[B25] Tinuper P. Bisulli F. Cross J. H. Hesdorffer D. Kahane P. Nobili L. (2016). Definition and Diagnostic Criteria of Sleep-Related Hypermotor Epilepsy. Neurology 86 (19), 1834–1842. 10.1212/WNL.0000000000002666 27164717 PMC4862248

[B26] Ulmer A. J. Scholz W. Ernst M. Brandt E. Flad H.-D. (1984). Isolation and Subfractionation of Human Peripheral Blood Mononuclear Cells (PBMC) by Density Gradient Centrifugation on Percoll. Immunobiology 166 (3), 238–250. 10.1016/S0171-2985(84)80042-X 6329947

[B27] Weckhuysen S. Marsan E. Lambrecq V. Marchal C. Morin-Brureau M. An-Gourfinkel I. (2016). Involvement of GATOR Complex Genes in Familial Focal Epilepsies and Focal Cortical Dysplasia. Epilepsia 57 (6), 994–1003. 10.1111/epi.13391 27173016

[B28] Zhang B. Zou J. Rensing N. R. Yang M. Wong M. (2015). Inflammatory Mechanisms Contribute to the Neurological Manifestations of Tuberous Sclerosis Complex. Neurobiol. Dis. 80, 70–79. 10.1016/j.nbd.2015.04.016 26003087 PMC4468035

