# Supplementary figures and images for: A Novel Loss-of-Function Mutation in the NPRL3 Gene Identified in Chinese Familial Focal Epilepsy with Variable Foci
Source: Front Genet. 2021 Nov 12;12:766354. doi: 10.3389/fgene.2021.766354 (PMC8633433; doi:10.3389/fgene.2021.766354)

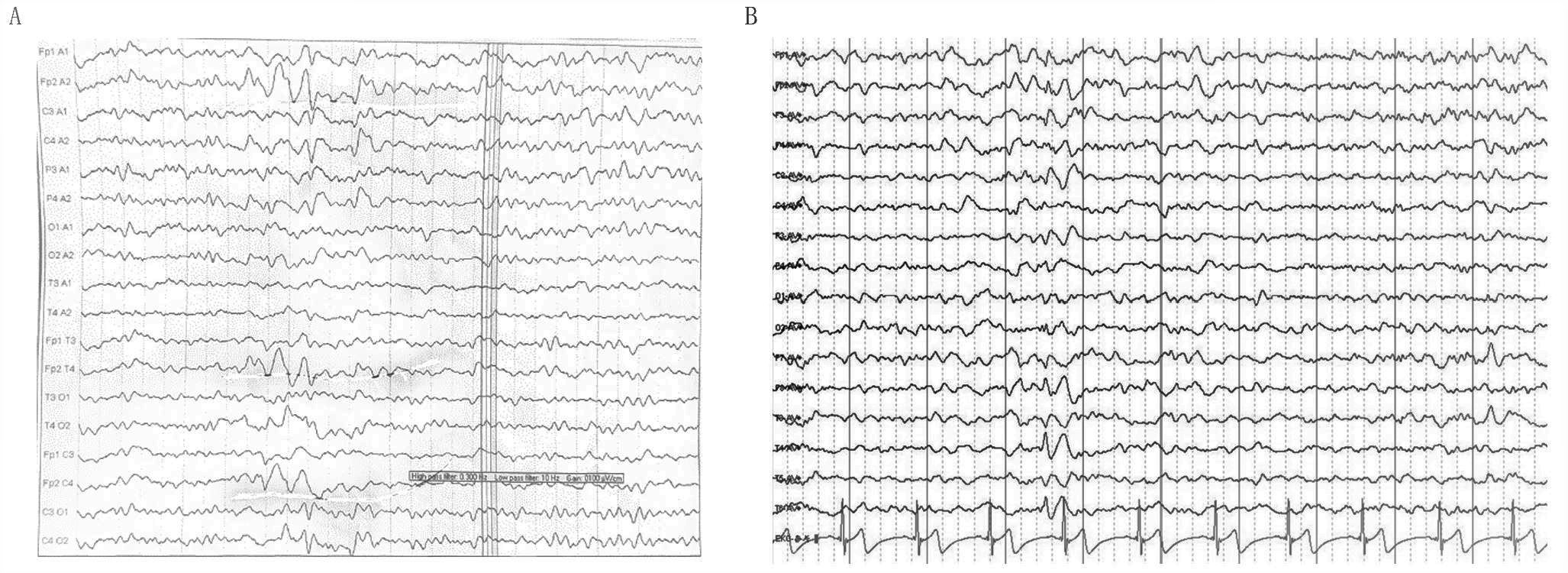

Supplement: Supplementary file 1 [file Image1.JPEG]
