# Supplementary material for: A Novel Loss-of-Function Mutation in the NPRL3 Gene Identified in Chinese Familial Focal Epilepsy with Variable Foci
Source: Front Genet. 2021 Nov 12;12:766354. doi: 10.3389/fgene.2021.766354 (PMC8633433; doi:10.3389/fgene.2021.766354)
Supplement: Supplementary file 2 [file DataSheet1.PDF]

Figure legend

FIGURE S1| (A) Interictal EEG of Proband VI-1 showed right frontal sharp wave. (B) Interictal EEG of IV-7 showed right temporal sharp and slow wave.
